# Use of MRI, metabolomic, and genomic biomarkers to identify mechanisms of chemoresistance in glioma

**DOI:** 10.20517/cdr.2019.18

**Published:** 2019-09-19

**Authors:** Cathy W. Levenson, Thomas J. Morgan, Pamela D. Twigg, Timothy M. Logan, Victor D. Schepkin

**Affiliations:** ^1^Department of Biomedical Sciences, Florida State University College of Medicine, Tallahassee, FL 32306, USA.; ^2^Program in Neuroscience, Florida State University, Tallahassee, FL 32306, USA; ^3^Department of Chemistry and Biochemistry and Institute for Molecular Biophysics, Florida State University, Tallahassee, FL 32306, USA.; ^4^Current Address: Department of Chemistry, University of Alabama in Huntsville, Huntsville, AL 35899, USA.; ^5^National High Magnetic Field Laboratory, Florida State University, Tallahassee, FL 32306, USA.

**Keywords:** Sodium MRI, diffusion, genes, resistance, glycolysis, warburg effect

## Abstract

Gliomas are the most common form of central nervous system tumor. The most prevalent form, glioblastoma multiforme, is also the most deadly with mean survival times that are less than 15 months. Therapies are severely limited by the ability of these tumors to develop resistance to both radiation and chemotherapy. Thus, new tools are needed to identify and monitor chemoresistance before and after the initiation of therapy and to maximize the initial treatment plan by identifying patterns of chemoresistance prior to the start of therapy. Here we show how magnetic resonance imaging, particularly sodium imaging, metabolomics, and genomics have all emerged as potential approaches toward the identification of biomarkers of chemoresistance. This work also illustrates how use of these tools together represents a particularly promising approach to understanding mechanisms of chemoresistance and the development individualized treatment strategies for patients.

## Introduction

Accounting for more than approximately 80% of all primary central nervous system malignancies, gliomas are the most common form of central nervous system tumor. They include a wide variety of brain tumors including oligodendrogliomas, ependymomas, and astrocytic tumors such as astrocytoma, anaplastic astrocytoma, and glioblastoma, as well as tumors of mixed population^[1]^. Glioblastoma multiforme (GBM), makes up over 60% of adult brain tumors, making it the most common, and most deadly, brain tumor^[2]^. GBM has a peak incidence between 55 and 60 years of age with a survival rate of only 14-15 months^[3]^. Survival times are even shorter for the GBM-related tumor gliosarcoma^[4]^.

While there are a number of new therapies currently in clinical trial^[5-7]^, the current standard of care includes surgery, radiation, and systemic therapy^[8-10]^. Maximum safe resection reduces tumor size and can improve quality of life. Post-surgical radiation therapy used to eliminate remaining cells, while not without side effects such as radiation necrosis and neuronal damage, increases life expectancy^[11,12]^. The addition of chemotherapy also improves survival. For GBM, the most commonly used drugs include temozolomide (TMZ), the current standard of care, 1,3-bis (2-chloroethyl)-1-nitrosourea (carmustine, BCNU), and lomustine (CCNU)^[1]^. Treatment of low grade tumors with procarbazine, lomustine, vincristine, or TMZ have been shown to improve survival^[13]^. Low-intensity alternating electrical fields of 200 Hz delivered via electrodes fixed directly to the scalp have been shown to disrupt mitosis, induce glioma cell cycle arrest, and improve survival times when combined with TMZ^[14]^.

Unfortunately, even when surgery, radiation, and chemotherapy can be used, high grade tumors inevitably return. The remaining cells develop genetic and metabolic adaptations that result in resistance to radiation and chemotherapy, enabling them to evade further treatment^[15-17]^. The development of chemoresistance results in highly aggressive cancer cells, rapid tumor regrowth, and inevitable patient death. In addition to acquired chemoresistance, cancer cells frequently have intrinsic resistance even before they have been exposed to drug treatment^[18]^.

Given the problems that both intrinsic and acquired resistance pose for treatment and survival, the need for reliable biomarkers of resistance is clear. Current work is actively examining the sensitivity and specificity of genomic, metabolic, and imaging biomarkers to identify and monitor the response to treatment and the development of chemoresistance leading to individualized treatment plans that maximize progression-free survival.

Currently, the three major types of biomarkers under investigation for detection and monitoring of chemoresistance in glioma are imaging biomarkers, acquired by magnetic resonance imaging (MRI), metabolomics to detect changes in cellular metabolism that confer resistance, and genomics to identify changes in gene expression that lead to molecular alterations that permit cancer cells to evade treatment. This article reviews the current progress in using these three approaches towards the goals of detecting and monitoring chemoresistance, understanding the mechanisms associated with chemoresistance, and using this information to design new, individualized therapies for the treatment of glioma.

## Glioma magnetic resonance imaging

MRI is an important tool for diagnosis of glioma as well as determination of the site and size of the tumor before and after surgical resection and treatment. Use of T1- and T2-weighted sequences and contrast-enhanced T1-weighted images is currently regarded as the gold standard^[19]^. Recent work however, is beginning to expand the use of MRI beyond diagnosis toward monitoring and predicting the response to therapy including the development of chemoresistance.

### Monitoring treatment response by MRI

Work is also being done to refine the use of magnetic resonance spectroscopy^[20,21]^, perfusion-weighted imaging^[22,23]^, diffusion-weighted imaging^[24,25]^, and the apparent diffusion coefficient (ADC)^[25-27]^ to predict the response to therapy^[19]^. For example, diffusion mapping after treatment shows an increase in water diffusion in the early stages after treatment that appears to be the result of apoptotic cell shrinkage and a loss of cell membrane integrity^[28,29]^.

Advances in sodium magnetic resonance imaging (Na-MRI) have also made it an attractive biomarker of treatment response. The rationale for the use of Na-MRI is based on the knowledge that intracellular sodium concentrations are around 15 mM, while extracellular concentrations are approximately 140 mM^[30]^. This means that not only can Na-MRI detect sodium heterogeneity within a tumor^[31]^, but also as tumor cells are damaged by radiation or chemotherapeutic treatment, there is an expected 1.4 to 1.8-fold increase in sodium^[30-33]^. Particularly noteworthy is the finding that changes in tumor sodium appear to precede shrinking of the tumor. In a pre-clinical model the sodium signal was significantly changed by day 4 post-treatment, while the tumor size did not markedly change until day 11^[30]^. This detection ability makes sodium MRI a potentially powerful tool as a non-invasive, early predictor of treatment efficacy^[30]^, particularly when coupled with proton imaging^[34,35]^.

Given the apparent power of Na-MRI for monitoring the response to treatment, it is not surprising that several clinical studies have now tested the possible use of Na-MRI at 3 T in patient populations with glioma. Together these studies have shown elevated total sodium levels in humans with glioma^[36-39]^. For example, normal white matter and putamen sodium measured in 8 untreated patients was determined to be approximately 30 mM and 35 mM, respectively. In contrast, tumor sodium concentrations were almost 60 mM^[39]^. Because the highest concentrations of sodium are normally found in the extracellular space, it was not initially clear if the elevations in tumor sodium concentration were the result of elevations in intracellular concentration, increases in the amount of extracellular space in the tumor, or some combination of the two. However, the most recent work in untreated patients suggests a 2-fold increase in extracellular or interstitial space of solid tumors^[39]^. Another study evaluated two patients with GBM at baseline (prior to treatment), 16-17 days after the initiation of treatment, and 72 days from the start of therapy with TMZ. Consistent with the pre-clinical work, chemotherapy increased tumor sodium. However, it was difficult to determine the extent to which this increase was due to a rise in intracellular sodium or the result of therapy-induced edema and necrosis^[31]^.

### Monitoring chemoresistance by MRI

Despite these advances in MRI-based biomarkers for the response to therapy, there is still a critical need for non-invasive imaging tools for detecting and monitoring the development of resistance to treatment. Initial reports showed that diffusion MRI was able to detect the loss of therapeutic response to BCNU in rodent 9L glioma cells implanted intracranially, suggesting that this method, which measures water mobility, can detect the development of chemoresistance^[40]^. Although difficult to quantify using this method, the work did suggest a reduced response to a second round of BCNU treatment, and opened the door for MRI-based tools to study chemoresistance.

The next advance resulted from the finding that Na-MRI was even more effective than diffusion at detecting the development of resistance following an initial round of therapy^[34]^. Rodent 9L glioma tumors, implanted subcutaneously, were treated with 26.6 mg/kg BCNU. As expected, at 9.4 T both proton and Na-MRI detected cell death and a reduction in tumor size over three weeks. However, when a second BCNU dose was administered to the same animals, a dramatically smaller diffusion response was detected. Na-MRI proved to be more sensitive to the change of tumor resistance^[34,41]^. While these data show that Na-MRI can detect the increase in chemoresistance after therapy, the long-term goal is to develop rapid, non-invasive MRI-based approaches that will not only detect chemoresistance after treatment, but to accurately predict chemoresistance prior to treatment.

Towards this goal, preclinical studies using ultra-high field MRI (21.1 T) have now shown that Na-MRI has the ability to detect chemoresistant tumors before the start of treatment (US Patent 8,880,146). An ultra-short echo time of 0.14 ms and high resolution 3D Na-MRI was able to detect differences between intracranial tumors that were generated from rodent glioma cell lines (9L gliosarcoma) with different levels of resistance to BCNU^[42]^. Tumors derived from drug-naïve BCNU-sensitive cells had a sodium concentration that was 173.4% ± 6.5%, relative to the surrounding normal brain tissue. In contrast, tumors derived from BCNU-resistant 9L cells had sodium concentrations that were 126.7% ± 7.5% of normal control concentrations (*P* < 0.001). The differences in diffusion between drug-sensitive and resistant tumors, while statistically different (*P* < 0.01), were not as robust as sodium (150.9% ± 5.7% *vs*. 140.1% ± 4.8%). Similarly, the time course of tumor diffusion and sodium concentration in the resistant cells showed that there was very little change in diffusion (1.2% ± 0.8% per day) while the change in sodium was 5.8% ± 0.8% per day. Thus, while diffusion MRI (ADC) was able to detect differences between the BCNU-resistant (9L-R) and BCNU-sensitive (9L-S) tumors, the sodium signal was a more robust biomarker of chemoresistance and able to detect a relatively small difference in sensitivity to treatment^[42]^. While this work has not yet been translated to a clinical population, these data raise exciting prospects for the use of Na-MRI clinically for the early detection of chemoresistance that could inform individualized treatment decisions, particularly now that high field strength MRI machines are being developed for clinical use. In fact, work is currently on-going to design and build MRI instruments between 14 and 20 T for human imaging^[43]^.

## Glioma metabolomics

The finding that the sodium signal is altered by chemoresistance raises questions about the role of sodium in glioma cell metabolism and mitochondrial function. It has long been recognized that cancer cells have altered metabolic profiles compared to normal cells^[44-48]^. While the highly proliferative nature of tumor cells requires them to generate nucleotides, lipids, proteins, and ATP, they also appear to have alterations in mitochondrial metabolism, leading to a reduced dependence on oxidative phosphorylation and a correspondingly higher dependence on glycolysis for the production of ATP even in the presence of adequate oxygen^[49,50]^. Glycolysis is the production of pyruvate and lactate from glucose, while oxidative phosphorylation utilizes the pyruvate from glycolysis to produce ATP in the mitochondria from tricarboxylic acid cycle intermediates. The increased glycolysis in cancer cells is known as the Warburg effect^[44,51,52]^, an effect that may play a role in the induction of treatment-resistant cancer stem cells^[53]^ and other resistance-related mechanisms.

The degree to which the Warburg effect participates in the development of chemoresistance in cancers such as glioma is controversial^[54,55]^ as clearly not all cancer cells have the same phenotype. Some work has identified TMZ-resistant glioma cells that have decreased glucose consumption, lactate production, and increased mitochondrial coupling compared to parental cells^[56,57]^, suggesting a reversal of the Warburg effect in chemoresistance. However, most work has reported data consistent with TMZ resistance being associated with an enhancement of the Warburg effect. For example, there is an increased expression of glucose transporters^[58]^ and glucose utilization^[53,59]^ in TMZ-resistant cells. Lactate dehydrogenase also enhanced resistance to both TMZ and radiation^[46]^.

### Evaluating chemoresistance by metabolomics

**Figure 1:**
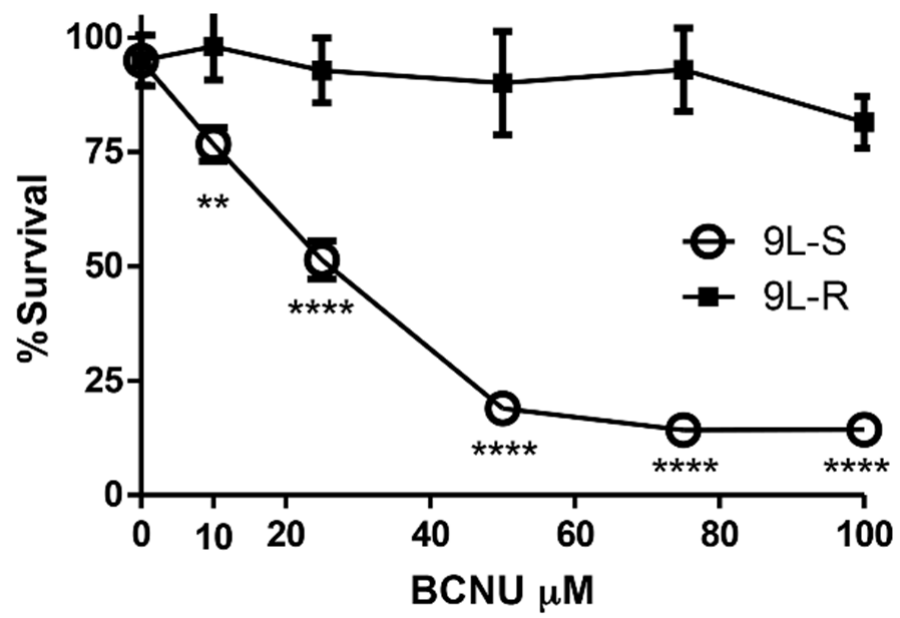
Development of BCNU-resistant 9L glioma cells. BCNU sensitive 9L glioma cells (9L-S) were grown in the presence of increasing concentrations of BCNU resulting in a 9L subculture that was BCNU-resistant (9L-R). Cell viability of 9L-S and 9L-R cells was quantified after exposure of both cell types to increasing concentrations of BCNU and expressed as percent survival (mean ± SD, *n* = 6). Significantly different from 9L-S at **P ≤ 0.01 and ****P ≤ 0.0001. BCNU: 1,3-bis (2-chloroethyl)-1-nitrosourea

One limitation of the work on chemoresistance in glioma is that much of this work has been conducted in TMZ–resistant cells. The contradictory data about the role of Warburg in chemoresistance, and the paucity of data in glioma cells resistant to chemotherapeutics other than TMZ led us to use metabolomics to evaluate metabolic changes in BCNU-resistant glioma cells and test the hypothesis that acquired BCNU resistance involves modifications to energy metabolism that enhance the Warburg effect. To test this hypothesis, we first generated a line of rat 9L glioma cells that are resistant to BCNU by repetitive dosing with increasing levels of the alkylating agent BCNU (10-100 µM). Maintenance of cultured 9L rat glioma cells with BCNU resulted in the selection of resistant cells. Figure 1 shows that resistant (9L-R) cells were significantly more resistant to BCNU than the parental, BCNU-sensitive line (9L-S) such that the half maximal inhibitory concentration (IC_50_) was 19 µM for the 9L-S parental line and 146 µM for the 9L-R line.

**Figure 2:**
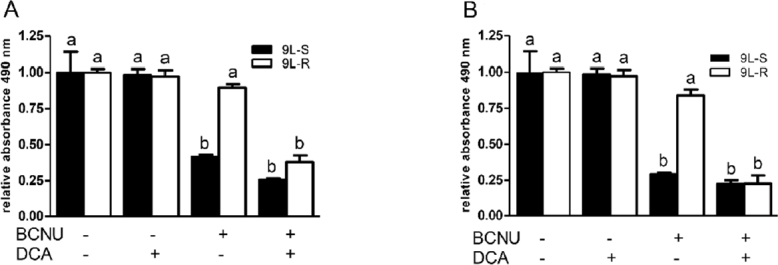
Dichloroacetate (DCA) improves the responsiveness of resistant glioma cells to BCNU. Treatment of BCNU-sensitive (9L-S) and BCNU-resistant (9L-R) glioma cells with (A) 75 µM or (B) 100 µM BCNU resulted in significant death of 9L-S, but not 9L-R cells. Addition of 25 mM DCA potentiated the action of BCNU in 9L-R cells at both concentrations of BCNU. Bars (mean ± SD, *n* = 6) with different letters (a *vs*. b) are significantly different from each other at *P* ≤ 0.05. DCA: dichloroacetate

Treatment with dichloroacetate (DCA) inhibits the enzyme pyruvate dehydrogenase kinase, activates pyruvate dehydrogenase, and increases oxidative phosphorylation. It has been used in combination with other drugs to relieve the Warburg effect in glioma and induce cell death^[60]^. Treatment with 25 mM DCA or BCNU alone did not reduce the viability 9L-R cells [Figure 2]. However, combining this dose of DCA with 75 µM [Figure 2A] or 100 µM [Figure 2B] BCNU resulted in significant reductions in cell viability. Releasing glioma cells from the Warburg effect enhances treatment-induced cell death - an effect that was more pronounced in chemoresistant cells. These data support the published literature suggesting a role for the Warburg effect in chemoresistance.

We then followed the metabolism of ^13^C-labeled glucose using gas chromatography-mass spectrometry (GC-MS) of cell extracts to compare the metabolic profile of BCNU-sensitive (9L-S) and BCNU-resistant (9L-R) cells. It has been well-established that the specific labeling pattern of key metabolites (called the isotopomer pattern) can be used to determine the relative activities of metabolic pathways contributing to the synthesis of those metabolites after correction for incorporation of the natural abundance isotope. The goal of this work was to establish a metabolomics approach that would permit the identification of key components of glucose metabolism that are altered in BCNU-resistant cells and provide additional insights into the mechanisms associated with acquired chemoresistance. This goal was supported by comparison of metabolic profiles in the presence or absence of DCA.

In brief, this metabolomic analysis was accomplished by first growing cells in the presence or absence of 75 or 100 µM BCNU and/or 25 mM DCA followed by a 24 h incubation with ^13^C-labeled glucose that were quenched with degassed, ice-cold 50% aqueous acetonitrile with 0.17 mg/mL norleucine, dried under vaccum overnight, redissolved in anhydrous pyridine, and derivatized by the addition of 20 µL N-methyl-N-(tert-butyldimethylsilyl)trifluoro-acetamide (TBDMS) containing 1% tert-butyldimethylchlorosilane (Thermo Scientific, Rockford, IL). Derivatized samples were injected in splitless mode into an HP Agilent 6890 series gas chromatograph coupled with an HP Agilent 5973 mass selective detector and separated on DB5-MS 30 cm × 250 µm × 0.25 µm column with a 10-cm guard column (J&W Scientific, Folsom, CA). Metabolites were identified by retention time and mass spectrum comparison with standards. The prevalence of particular mass isotopomers (species having the same isotopic mass) for a given compound provides information about the incorporation of labeled media components into metabolites. A mass isotopomer distribution vector (MID) was calculated for the molecular and fragment ions for assigned observed metabolites using in-house scripts written in the programming language^[61]^. Data were analyzed using student’s *t*-test or one way ANOVA with either Tukey’s or Bonferroni post-hoc tests and considered significantly different at *P* ≤ 0.05.

### Resistance to BCNU alters glucose metabolism

**Figure 3:**
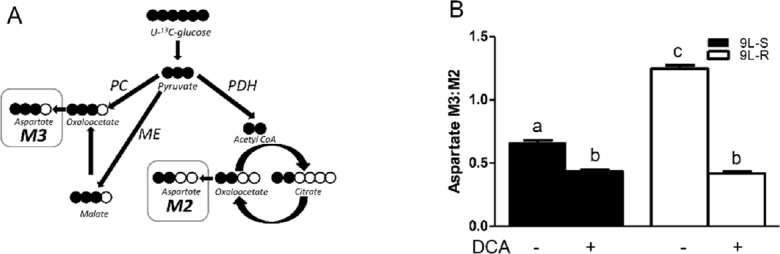
Effect of chemoresistance on aspartate isotopomer ratios. A: Model depicting the pathways for aspartate M2 and M3 isotopomer production from U-^13^C glucose; B: Aspartate M3:M2 isotopomer ratios in BCNU-sensitive (9L-S) and BCNU-resistant (9L-R) glioma cells in the absence and presence of 40 mM DCA. Bars (mean ± SD, *n* = 6) with different letters are significantly different from each other at *P* ≤ 0.05. PC: pyruvate carboxylase; PDH: pyruvate dehydrogenase; ME: malic enzyme

Comparison of the M3:M2 ratio of TCA cycle intermediates permited the evaluation of cellular metabolism including metabolism of pyruvate via anaplerotic conversion to oxaloacetate or malate versus the pyruvate dehyrogenase/citrate synthase pathway (PDH/CS)^[62-64]^. Oxaloacetate levels were below the limit of detection in the GC-MS experiments but aspartate consistently showed an intense signal and was used as a proxy for TCA cycle intermediates [Figure 3A]. As shown in Figure 3B, the anaplerotic reactions accounted for approximately 40% of pyruvate metabolized into TCA cycle intermediates in 9L-S cells (M3:M2 ratio of approximately 0.6), but acquisition of BCNU resistance increased the anaplerotic contribution to about 55%. Treatment with DCA reduced the aspartate isotopomer ratio in both cell types to approximately 0.5 [Figure 3B], corresponding to a 2:1 ratio of PDH/CS activity versus the anaplerotic reactions.

**Figure 4:**
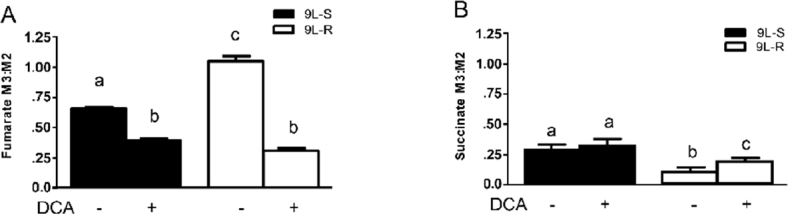
Effect of chemoresistance on fumarate and succinate isotopomer ratios. Isotopomer ratios for (A) fumarate and (B) succinate in BCNU-sensitive (9L-S) and BCNU-resistant (9L-R) glioma cells in the absence and presence of 40 mM DCA. Bars (mean ± SD) with different letters are significantly different from each other at *P* ≤ 0.05

The chemoresistance-induced reorganization of pyruvate metabolism and the corresponding effect of DCA treatment was also evident from the isotopomer patterns observed in other TCA cycle intermediates. For instance, the M3:M2 ratio for fumarate was significantly higher in the resistant cells than in the 9L-S cells, and treatment with DCA nearly equalized this ratio [Figure 4A]. On the other hand, the succinate M3:M2 ratio was significantly smaller than that of fumarate in 9L-S cells, such that carbons entering the TCA cycle via anaplerotic reactions accounted for only about 20% of the total succinate pool [Figure 4B]; DCA treatment had essentially no effect on this. By comparison, anaplerotic reactions contributed even less to the succinate pool in resistant cells (approximately 10%) and DCA treatment almost doubled this value.

These data show that pyruvate was primarily metabolized by the TCA cycle through pyruvate dehydrogenase and citrate synthase in 9L-S cells, but that anaplerotic reactions of pyruvate carboxylase and/or malic enzyme provided the primary route in the 9L-R cells. Our method was unable to determine whether the higher M3:M2 ratio observed for aspartate, malate, and fumarate in the 9L-R cells resulted from an actual increase in pyruvate carboxylase activity, from a decrease in PDH activity, or from a combination. Regardless, the increased M3:M2 ratio observed for these compounds in 9L-R cells shows that acquired chemoresistance increases the relative activity of pyruvate metabolized through the anaplerotic reactions and decreases the dependence of the PDH pathway. The reduced activity of pyruvate dehydrogenase is typically seen in cancer cells and our data suggest that acquisition of BCNU resistance exacerbates this effect by creating a more Warburg-like state compared to drug-sensitive glioma cells.

Further support for this hypothesis was obtained by treating 9L-S and 9L-R cells with DCA. As mentioned above, DCA is known to activate pyruvate dehydrogenase, reversing tumor-specific modifications in mitochondrial physiology, both *in vivo* and *in vitro*^[65]^. We showed that BCNU treatment reduced cell viability of 9L-R cells in the presence of DCA, but not in its absence. DCA treatment also reduced the M3:M2 ratio of aspartate, malate, and fumarate, indicating a relative reduction in anaplerotic activity and a corresponding increased activity of the PDH/CS reactions. Again, DCA reduced the M3:M2 ratio to essentially the same level in BCNU-sensitive and -resistant cells. Thus, our data suggest that the ability of DCA to re-sensitize the 9L-R cells to BCNU may be a consequence of reversal of the Warburg effect.

### Resistance to BCNU alters amino acid metabolism

**Figure 5:**
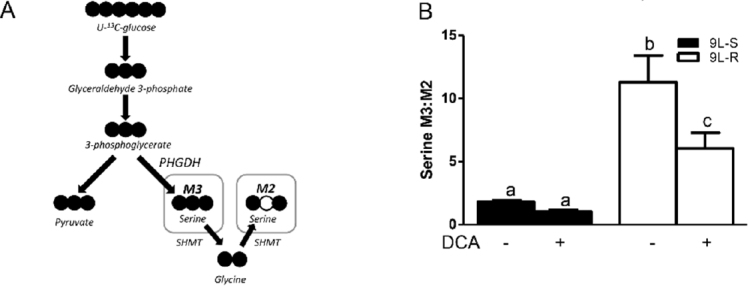
Effect of chemoresistance on serine isotopomer ratios. A: Model depicting the pathway for serine M2 and M3 isotopomer production from U-^13^C glucose; B: Serine M3:M2 isotopomer ratios in BCNU-sensitive (9L-S) and BCNU-resistant (9L-R) glioma cells in the absence and presence of 40 mM DCA. Bars (mean ± SD) with different letters are significantly different from each other at *P* ≤ 0.05

**Figure 6:**
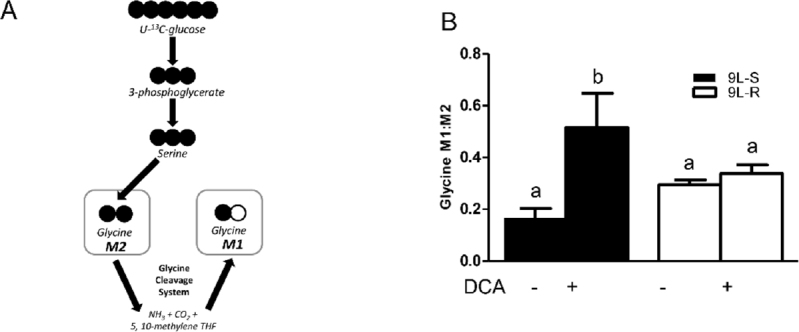
Effect of chemoresistance on glycine isotopomer ratios. A: Model depicting the pathway for glycine M1 and M2 isotopomer production from U-^13^C glucose; B: Glycine M1:M2 isotopomer ratios in BCNU-sensitive (9L-S) and BCNU-resistant (9L-R) glioma cells in the absence and presence of 40 mM DCA. Bars (mean ± SD) with different letters are significantly different from each other at *P* ≤ 0.05

The development of BCNU-resistance also had a significant effect on the isotopomer patterns of serine and glycine. The M3 and M2 isotopomers of serine reflect the relative amount synthesized from 3-phosphoglycerate (the M3 isotopomer) versus that synthesized from glycine (the M2 isotopomer) [Figures 5 and 6]. As shown in Figure 5B, the serine M3:M2 ratio in 9L-S cells was approximately 2. Treatment with DCA slightly reduced this ratio. Chemoresistant cells had a significantly higher serine M3:M2 ratio (*P* < 0.05). Again, DCA treatment reduced this ratio, but not to the extent seen in 9L-S cells [Figure 5B]. Additionally, while resistance did not change the glycine isotopomer ratio, the response of glycine metabolism to DCA treatment can distinguish between BCNU-sensitive and -resistant cells [Figure 6].

As shown in Figure 5A, serine is synthesized from the glycolytic intermediate 3-phosphoglycerate (3-PG) in 3 steps with the rate primarily controlled by the activity of the first enzyme in this pathway, phosphoglycerate dehydrogenase (PHGDH); the M3 isotopomer derives from ^13^C_3_-3PG. Serine is converted to glycine, with transfer of the hydroxymethyl carbon to tetrahydrofolate (THF), resulting in ^13^C_2_-glycine and ^13^C-labeled N^5^,N^10^-methylene THF (^13^C-CH_2_THF). This reaction is catalyzed by serine hydroxymethyl transferase (SHMT). When the SHMT reaction operates in the reverse direction, serine can be labeled with two or three ^13^C atoms, depending on the labeling of CH_2_THF and glycine. Thus, the M3:M2 ratio in serine can be interpreted in terms of the relative activity of the “forward” synthesis of serine from 3-PG versus that of the “reverse” reaction, or synthesis of serine from glycine by SHMT. Alternatively, it can be interpreted in terms of the availability of ^13^C-CH_2_THF for conversion of glycine into serine. In the BCNU-sensitive cells, the M3:M2 ratio is approximately 2 but is increased significantly to > 10 in BCNU-resistant cells. This could indicate a significant decrease in the reverse reaction of SHMT in the resistant cells, or a significant reduction in the concentration of ^13^C-CH_2_THF. Many studies have shown that acquired and intrinsic resistance is associated with aberrant patterns of hypermethylation of DNA catalyzed by DNA methyltransferase^[65-67]^. The methyl donor in this reaction is S-adenosyl methionine, whose synthesis is regulated by cellular levels of CH_2_THF. Thus, future work will be needed to determine if the observed changes in serine isotopomer pattern reflect alterations in DNA methylation in BCNU-resistant cells.

In summary, while clearly not all resistance is the result of the Warburg effect, much of the data in the literature support the hypothesis that an enhancement of the Warburg effect is associated with acquired chemoresistance. Furthermore, our work with BCNU-resistant cells line reported here adds to the existing literature on TMZ-resistance by identifying several metabolic changes associated with acquired resistance in glioma cells. More importantly, however, this work illustrates the strength of a metabolomic approach for potentially phenotyping glioma, evaluating resistance, and identifying metabolic pathways that may serve as future targets for overcoming chemoresistance and developing novel treatments for chemoresistant tumors.

## Glioma genomics

A large number of studies have established the association between wide-spread genomic changes and glioma tumorigenesis. Evaluation of gliomas at the genomic level is crucial because histologically identical tumors have been shown to have very different genomic alterations^[68,69]^. The four genetic alterations in glioma that are most commonly utilized for diagnostic purposes are MGMT promoter methylation status, deletion of chromosomes 1p/19q, isocitrate dehydrogenase mutations, and BRAF duplications or fusions^[70]^. Other common genomic alterations include alterations in expression or mutations in epidermal growth factor receptor, platelet-derived growth factor receptor (PDGFRα)^[48,59]^, members of the PI3K/AKT/mTOR pathway^[48,53,59]^, lactate dehydrogenase^[48]^, p53^[48,71]^, PTEN, MYB, and MYC^[47,69]^.

### Genomic changes associated with chemoresistance to temozolomide

Like metabolomic approaches, most of the work using genomic approaches to determine the mechanisms responsible for chemoresistance has been conducted in TMZ-resistant cells by comparing them to TMZ-sensitive cells. These cells are characterized by chromosomal instability, alterations in MDM2, ERK, AKT, STAT3, ABC transporters, and PIK3 pathway members^[72,73]^, epigenetic regulation of the DNA repair enzyme MGMT^[58,68]^, as well as alterations in MSH6^[70]^ and aldehyde dehydrogenase^[74]^. Many of the genes that have been identified to date appear to play a role in the metabolic changes associated with chemoresistance. For example, over-expression of the gene that codes for the cytosolic version of isocitrate dehydrogenase (IDH1) has been shown in resistant glioma cells^[75]^. This enzyme converts isocitrate to the α-ketoglutarate that participates in the regulation of gene expression via histone methylation. Rather than being merely an adaptive response to rapid cell proliferation, this appears to be a selective response following exposure to chemotherapy^[75]^.

Hypoxia-related genes have also been implicated in the metabolic changes associated with chemoresistance and prognosis^[76]^. Specifically, the hypoxia-induced transcription factor HIF-1α has been linked to chemoresistance through several different molecular mechanisms. First, HIF-1α up-regulates the glucose transporters GLUT1 and GLUT3, both of which act in glioma cells to increase cellular glucose uptake. Consistent with the Warburg hypothesis, HIF-1α also regulates the glycolytic enzymes, hexokinase^[48]^, pyruvate kinase, and lactate dehydrogenase^[53]^. HIF-1α has also been shown to up-regulate vascular endothelial growth factor (VEGF) that plays a role in vascularization of tumors^[77]^. Additionally, deep sequencing of RNA has identified a large number of novel microRNAs associated in GBM tumors. Of these, miR-210-3p (which increases the transcriptional activity of HIF-1α and its target gene vascular endothelial growth factor), miR-24, and miR-125b have all be implicated in the development of chemoresistance^[77,78]^.

### Genomic changes associated with chemoresistance to BCNU

The alkylating agent BCNU has been infused into biodegradable wafers for implantation after surgical resection. The clinical outcomes of this approach have been mixed^[79]^, in part due to the rapid development of BCNU-resistance by high grade tumors. However, recent work has shown that BCNU application can be a safe and effective first-line treatment, particularly in patients with a methylated MGMT promoter^[80]^ or low miRNA-181d^[81]^. Limited work on genomic changes associated with BCNU resistance has shown that a link between BCNU and the increased expression of the *CLOCK1* gene may contribute to BCNU resistance through the AMPK/mTOR/HIF-1α pathway that regulates glycolysis^[82]^.

Because of the paucity of genomic data on chemoresistance to BCNU, we used rat 9L cells and microarray analysis to compare the gene expression profiles of BCNU-sensitive (9L-S) and BCNU-resistant cells and identify significant changes in gene expression associated with BCNU-resistance. Resistant cells were produced by continuous culture in increasing concentrations of BCNU as previously described producing two lines of resistant cells, 9L-R1 treated with 150 µM BCNU and 9L-R2 treated with 225 µM BCNU in culture. Additionally, 1 × 10^5^ 9L-S cells were implanted to produce an intracranial tumor in adult Sprague Dawley rats. After 11 days of tumor growth and confirmation by MRI, rats were treated with 26.6 mg BCNU/kg body weight. Following tumor regrowth, tumors were excised and cultured to propagate an additional line of resistant cells (9L-R3). Total cellular RNA was collected from all four cell lines and analyzed using a Roche/Nimblegen 12-plex microarray. Data analysis was completed with ArrayStar software (DNASTAR, Madison, WI). Expression patterns were normalized using the quantile normalization method and mRNA abundance were considered differentially regulated when there were changes in abundance of at least 1.5-fold with *P*-values ≤ 0.05. Pathway analysis was completed using www.pantherdb.com. Additionally, specific information about differentially regulated genes was obtained from www.ncbi.nlm.nih.gov/gene.

**Figure 7:**
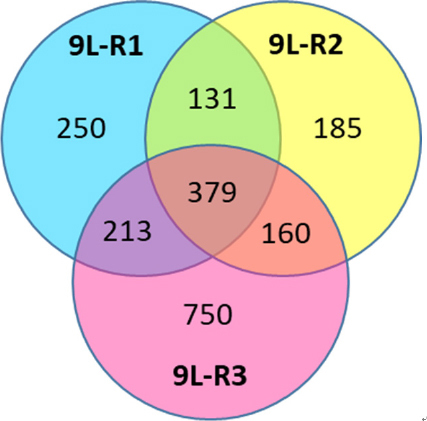
Effect of BCNU exposure to 9L rat glioma cells. Cells were exposed to 150 µM (9L-R1) or 225 µM (9L-R2) in culture or *in vivo* (9L-R3) after intracranial implantation (26.6 mg/kg body weight). RNA was subjected to 12-plex microarray to determine changes in gene expression compared to drug naïve cells (9L-S). Venn diagram illustrates the number of genes differentially regulated (*P* ≤ 0.05) in each of the three BCNU-resistant cell lines

Consistent with the literature on genomic changes in rapidly dividing cancer cells, each of the resistant 9L cell lines had its own distinct genomic pattern. Comparison of the resistant line to the parent 9L-S line identified 379 genes that were differentially regulated in all three of the resistant lines [Figure 7]. Of these 275 had expression levels that were below 50% of control (9L-S) values and 104 had expression levels that were > 150% of control. Genes of particular mechanistic interest are shown in Table 1. Like TMZ-resistance, these data showed that BCNU-resistance is associated with significant increases in DNA repair enzyme MGMT^[59,69]^. Of particular interest was the finding that nerve growth factor (the NGF) and its receptor were both significantly down-regulated in resistant cells. This finding is important for our understanding of the mechanisms of resistance as a recent report has shown that NGF prevents glioma proliferation via receptor-mediated pathways^[83]^. This suggests that the significant down-regulation of both ligand and receptor in resistant cells may play a role in the aggressive nature of resistant cells as well in their ability to proliferate in the presence of BCNU.

**Table 1:** BCNU-Resistance Genes

Gene	Percent of Expression in Drug Naïve 9L-S				
	9L-R1	9L-R2	9L-R3	mean ± SD	*P*-value
CRABP1	0.97	0.97	2.25	1.40 ± 0.74	9.7 × 10^-12^
NGF receptor	3.99	3.99	3.56	2.43 ± 1.51	1 × 10^-8^
NGF	8.26	7.59	8.55	8.13 ± 0.49	2 × 10^-9^
Brain creatine kinase	9.07	8.67	9.65	8.87 ± 0.28	2.7 × 10^-10^
VG Na channel, type 1	27.7	13.9	17.5	19.70 ± 7.16	1.5 × 10^-6^
Na/Ca exchanger 5	35.7	44.9	39.7	40.1 ± 4.61	2.9 × 10^-6^
FGF 9	423	818	275	505 ± 280	3.1 × 10^-14^
TGF-β2	508	849	772	710 ± 179	4.5 × 10^-11^
MGMT	850	642	652	715 ± 117	1.1 × 10^-8^
Galectin 3	3055	311	3342	2236 ± 1673	5.1 × 10^-10^

Microarray analysis was used to identify genes regulated in chemoresistant lines (9L-R1, 9L-R2, and 9L-R3) compared to the parent drug naïve line of 9L cells. NGF: nerve growth factor; VG Na channel; voltage-gated sodium channel; Na/Ca exchanger; sodium/calcium exchanger; FGF: fibroblast growth factor; TGF: transforming growth factor; MGMT: O-6-methylguanine-DNA methyltransferase

Another potentially important mechanism is the finding that cellular retinoic acid binding protein-1 (CRABP1) was the most intensely down-regulated mRNA in the three resistant lines. While this gene has not been previously reported to be regulated in glioma cells, CRABPI has been shown to play a role in the induction of apoptosis in a mouse ovarian epithelial cancer cell line treated with retinoic acid^[84]^. Down-regulation of CRABPI in chemoresistant glioma suggests a role for this mediator of retinoic acid in glioma survival. Galectin 3 was the second most highly regulated mRNA in this data set. Galectin 3 has previously been shown to be up-regulated in human GBM where it contributes to cell survival^[85]^. The data reported here suggest that inhibition of galectin 3 could serve as a powerful target for overcoming chemoresistance. Brain creatine kinase expression has previously been shown to be lower in GBM than in surrounding normal tissue^[86]^. The finding reported in Table 1 that this mediator of energy metabolism is even lower in BCNU resistant glioma (< 10% of drug naïve 9L-S cells) suggests a role for this gene in the survival of resistant cells. Finally, the finding that the type 1 voltage-gated sodium channel was significantly decreased in resistant cells is consistent with our previously reported Na-MRI data, reviewed here, showing that total sodium was significantly reduced in tumors derived from resistant 9L glioma cells compared to drug naïve tumors^[42]^.

## Conclusions

In conclusion, each of the three approaches to identifying biomarkers of chemoresistance come with specific strengths. Metabolomics and genomics have the potential to identify specific cellular and molecular mechanisms that are responsible for both intrinsic resistance as well as therapy-induced resistance. These mechanisms have the potential to serve as novel targets for reducing chemoresistance, but require surgical intervention or biopsy to obtain tissue. Imaging biomarkers, particularly those utilizing Na-MRI, have the advantage of being rapid, non-invasive, and easily employed before and after treatment. Ultimately, significant advances in treatment are most likely to come from a combination of these approaches. For example, non-invasive imaging-based biomarkers that can be correlated to changes in biochemical mechanisms would enable MRI to be used to collect cellular and molecular information non-invasively in patients with glioma. Thus, used together these powerful tools could inform treatment decisions, facilitate individualized treatment plans, and improve survival time in patients with gliomas.

## Declarations

### Copyright

© The Author(s) 2019.
